# Regulation of metabolism by the Mediator complex

**DOI:** 10.1007/s41048-016-0031-6

**Published:** 2016-11-01

**Authors:** Dou Yeon Youn, Alus M. Xiaoli, Jeffrey E. Pessin, Fajun Yang

**Affiliations:** 1 Division of Endocrinology, Department of Medicine, Diabetes Research Center, Albert Einstein College of Medicine, Bronx, NY 10461 USA; 2 Department of Molecular Pharmacology, Albert Einstein College of Medicine, Bronx, NY 10461 USA; 3 Department of Developmental and Molecular Biology, Albert Einstein College of Medicine, Bronx, NY 10461 USA

**Keywords:** Transcription, Mediator, Cofactor, Metabolism, Insulin resistance, Obesity

## Abstract

The Mediator complex was originally discovered in yeast, but it is conserved in all eukaryotes. Its best-known function is to regulate RNA polymerase II-dependent gene transcription. Although the mechanisms by which the Mediator complex regulates transcription are often complicated by the context-dependent regulation, this transcription cofactor complex plays a pivotal role in numerous biological pathways. Biochemical, molecular, and physiological studies using cancer cell lines or model organisms have established the current paradigm of the Mediator functions. However, the physiological roles of the mammalian Mediator complex remain poorly defined, but have attracted a great interest in recent years. In this short review, we will summarize some of the reported functions of selective Mediator subunits in the regulation of metabolism. These intriguing findings suggest that the Mediator complex may be an important player in nutrient sensing and energy balance in mammals.

## Introduction

Gene transcription in eukaryotic cells is orchestrated through extremely complicated processes and multiple steps, including initiation, elongation, and termination, with the initiation being the most studied regulation step of gene expression. Disturbance of transcription initiation often results in serious diseases, such as cancer, in humans (Lee and Young 2013). Among the numerous different components in the initiation step of eukaryotic transcription, RNA polymerase II (Pol II) and the general transcription factors (TFIIB, TFIID, TFIIE, TFIIF, and TFIIH) constitute the basal transcription machinery, and specific sets of transcription factors are essential to determine the activation or repression of the target genes. However, it is generally believed that most of the transcription factors in eukaryotes are unable to directly interact with Pol II, which is responsible for the production of all mRNAs. Studies in the past several decades suggest that this crucial gap is often filled by various transcription cofactors, which critically regulate the activation or repression in gene expression.

There is no doubt that human beings are currently experiencing an epidemic of obesity. As a result, the prevalence of type 2 diabetes (T2D) has sharply increased in the past decades. Dysregulation of genes in the pathways controlling glucose and/or lipid metabolism is common in states of insulin resistance and T2D, especially when patients are also diagnosed with non-alcoholic fatty liver disease (NAFLD) or cardiovascular disease (Brown and Goldstein 2008; Oh et al. 2013). The DNA-binding transcription factors and their cofactors have been one focus of the studies in the hope of understanding the molecular mechanisms that cause metabolic diseases. A few notable examples of transcription factors that regulate glucose and/or lipid metabolism include cAMP-regulated enhancer-binding protein (CREB), forkhead O box proteins (FoxOs), glucocorticoid receptor (GR), hepatic nuclear factors (HNFs), sterol response element binding protein-1c (SREBP-1c), carbohydrate response element binding protein (ChREBP), liver X receptors (LXRs), and peroxisome proliferator-activated receptor-γ (PPARγ) (Brown and Goldstein 2008; Oh et al. 2013; Lefterova et al. 2014). Among the transcription cofactors, a multi-subunit protein assembly called the Mediator complex has been linked to several of these transcription factors (Malik and Roeder 2010; Taatjes 2010; Conaway and Conaway 2011; Allen and Taatjes 2015). Here, we summarize the current understanding on the regulation of transcription factors by individual subunits of the Mediator complex, focusing mainly on the metabolic involvement of the mediator subunits in mammals.

### The Mediator complex as a transcription cofactor

The Mediator complex is able to bind to various transcription factors and integrates the transcriptional signals to the basal transcription machinery (Malik and Roeder 2010; Taatjes 2010; Conaway and Conaway 2011; Allen and Taatjes 2015). Originally discovered in yeast as a transcription cofactor (Kelleher et al. 1990; Flanagan et al. 1991; Kim et al. 1994), the mammalian Mediator complex has been given several different names in the early literature, including TRAP (thyroid hormone receptor-associated protein) (Fondell et al. 1996), ARC (activator-recruited cofactor) (Naar et al. 1999), or DRIP (vitamin D receptor-interacting protein) (Rachez et al. 1998). In HeLa cells, the Mediator complex can be biochemically isolated in at least two forms: the small mediator, also known as the core mediator (with a mass of about 600 kDa), that comprises up to 26 subunits organized into the head, middle, and tail sub-modules, and the large mediator (with a mass of about 1.2 MDa), which additionally contains the kinase sub-module of four subunits—cyclin-dependent kinase 8 (CDK8), cyclin C (CycC), MED12, and MED13, but lacks the MED26 subunit (Taatjes et al. 2002). However, in other studies only the large Mediator complex was observed in the biochemical purifications (Wang et al. 2001; Sato et al. 2004). Nevertheless, it is generally believed that the Mediator complex is heterogeneous both structurally and functionally. In terms of functions, initial data suggested that the small mediator functions to activate transcription initiation in vitro, while the large mediator is inactive or acts as a transcription repressor (Taatjes et al. 2002). However, the large mediator has also been linked to transcription elongation (Donner et al. 2010). In addition, the mediator kinase module behaves in a context-specific manner to either repress or activate transcription, depending on the transcription factors and/or target gene promoters (Nemet et al. 2014).

Since the Mediator complex can directly bind to Pol II (Naar et al. 1999; Casamassimi and Napoli 2007; Taatjes 2010; Soutourina et al. 2011; Lariviere et al. 2012), collectively it may regulate many genes. However, studies on individual subunits of the Mediator complex revealed remarkable transcription factor and/or tissue-specific functions for certain subunits. One example is that the MED15 subunit binds to and regulates SREBP-dependent transcription, while the MED25 subunit primarily controls VP16-mediated transcription (Mittler et al. 2003; Yang et al. 2004, 2006). Although it is unclear how many transcription factors could physically interact with the Mediator complex, it is less likely that all transcription factors need this complex to regulate target gene expression. For instance, currently there is no evidence that CREB or Myb can directly bind to the Mediator complex, and depletion of some subunits such as MED15 or MED25 had no effect on Myb-dependent gene transcription in HEK293 cells (Yang et al. 2004, 2006). Moreover, the mediator subunit abundance and/or subcellular location may be different under different conditions. For example, feeding, obesity, NAFLD, and aging can cause a significant reduction of CDK8 and CycC proteins in the liver due to mTORC1 activation (Feng et al. 2015). Another example is that in response to stress, CycC undergoes translocation from the nucleus to the cytoplasm to regulate mitochondrial fission (Cooper et al. 2014; Wang et al. 2015). Although little is known about how the mediator subunit abundance is regulated, it may alter the Mediator complex composition and thus may have profound effects on the mediator-dependent functions.

Recent structure studies have demonstrated the presence of many different conformational assembly states of the Mediator complex (Tsai et al. 2014; Wang et al. 2014), indicating the intrinsic flexibility and heterogeneity. Moreover, the functions of the Mediator complex are now expanded to include the transcription elongation (Donner et al. 2010; Takahashi et al. 2011) and termination (Mukundan and Ansari 2011), and mRNA processing (Huang et al. 2012) and export (Schneider et al. 2015). Due to its massive size and complexity, however, the precise molecular mechanism(s) by which the Mediator complex regulates the gene-specific transcription remain poorly understood but are likely to play important functional roles in both normal physiological and pathophysiological states.

### The head module

The head module includes MED6, MED8, MED11, MED17, MED18, MED20, MED22, MED27, MED28, MED29, and MED30. This sub-module maintains the overall structure of the Mediator complex. The importance of the head module has been demonstrated from studies showing that yeast loss of MED17 prevents nearly all mRNA synthesis (Holstege et al. 1998; Thompson and Young 1995). Although it is unclear whether MED17 plays a similarly essential role in mammalian cells, a recent study reported that MED17 in the liver regulates lipogenic gene expression and lipid metabolism through the LXR transcription factors (Kim et al. 2015), providing a mechanism for the mediator regulation of lipid metabolism. Moreover, MED17 also binds to VP16, p53, and the estrogen receptor (ER) (Burakov et al. 2000; Park et al. 2003; Mehta et al. 2009; Meyer et al. 2010; Kumafuji et al. 2014). Similarly, PPARγ interacts with both MED1 (Zhu et al. 1997; Yuan et al. 1998; Ge et al. 2008) and MED14 (Grontved et al. 2010) subunits of the middle and tail modules, respectively. Thus, it appears that a given transactivation domain sometimes recruits the Mediator complex by binding to more than one subunit.

It is the current understanding that the head and middle modules may bind to either Pol II or the kinase module in an exclusive manner (Knuesel et al. 2009a; Naar et al. 2002). The head module is thought to provide the greatest impact in controlling overall transcription primarily by shifting the transcription machinery from active to inactive state rather than serving as a binding site for other transcriptional regulators. The electron microscopy-derived structure and affinity pull-down experiments have identified MED17 as the main subunit that directly interacts with Pol II and promotes the transcription initiation of selected genes (Soutourina et al. 2011). Interestingly, the head module subunit MED30 is a metazoan-specific subunit, and a missense mutation of MED30 in mice resulted in a pleiotropic decrease in transcription of cardiac genes that are necessary for oxidative phosphorylation and mitochondrial integrity (Krebs et al. 2011). The mutation effect of MED30 can be partially protected by a ketogenic diet through increasing the expression of genes, such as *Pgc1a* and *Sod2* (Krebs et al. 2011). This study suggests a critical role of MED30 in metabolism, but the regulatory mechanism(s) shall be investigated in the future.

### MED1 provides a docking surface for nuclear receptors

The middle module includes subunits of MED1, MED4, MED7, MED9, MED10, MED19, MED21 MED26, and MED31. The MED26 subunit is a specific subunit of the small mediator (Taatjes et al. 2002) and it is required for transcription elongation (Takahashi et al. 2011). The most studied subunit of the middle module is MED1, but metabolic functions of other subunits in the middle module remain to be investigated.

MED1 is able to interact with numerous transcription factors or cofactors, including PPARα, PPARγ, GR, C/EBPβ, and PGC1α, which are all implicated in metabolic regulation (Zhu et al. 1997; Yuan et al. 1998) (See Jia et al. 2014 for a recent review on MED1). Although deletion of MED1 results in embryonic lethality at E11.5, the MED1 conditional knockout or mutant mice play an important role in adipogenesis through PPARγ, fatty acid oxidation through PPARα, mammary gland development through ER, liver steatosis through GR and constitutive androstane receptor (CAR), thermogenesis regulation through uncoupling protein 1 (UCP1) up-regulation, skeletal muscle function, and insulin signaling (Ge et al. 2002; Jia et al. 2004, 2009; Jiang et al. 2010; Iida et al. 2015).

Similar to the steroid receptor co-activator (SRC) and PGC1 families of transcription cofactors, MED1 contains two LXXLL motifs [located within amino acid (aa) 589–593 and 630–634, respectively] that provide the binding surfaces for various nuclear receptors, and either one of the LXXLL motifs is sufficient for protein–protein interactions (Chang et al. 1999; Ge et al. 2008). A dominant negative form of MED1 with mutant LXXLL motifs reduces the transcription activity of nuclear receptors and suppresses PPARγ-induced adipogenesis (Ge et al. 2002). However, these LXXLL motifs are not required for MED1 regulation of PPARγ in cultured MEFs, suggesting MED1 regulation of gene transcription through alternative mechanisms in a context-dependent manner. Moreover, the conserved N-terminus (aa1–530) of MED1 is also important for PPARγ-target gene expression (Ge et al. 2008). Deletion of MED1 in mouse liver abrogates PPARα-activated peroxisomal proliferation (Jia et al. 2004) and acetaminophen-induced hepatotoxicity through CAR (Jia et al. 2005). Moreover, liver-specific knockout of MED1 protects mice from excessive fat accumulation under high-fat diet, whereas the wild-type mice exhibited fatty liver (Bai et al. 2011).

Recently, MED1 has been found to interact with PRDM16, a key inducer of brown adipose tissue (BAT)-selective genes (Harms et al. 2015; Iida et al. 2015). Directly interacting with the N-terminus of MED1, PRDM16 promotes *Ucp1* gene expression in brown adipocytes (Iida et al. 2015). ChIP-Seq and ChIP-qPCR analyses show that MED1 is recruited to the enhancer sites of BAT-selective genes such as *Ucp1*, *Cidea*, *Ppara*, and *Pgc1a* in the wild-type, but not in PRDM16 knockout brown adipocytes (Harms et al. 2015). Furthermore, skeletal muscle-specific knockout of MED1 increased gene expression of *Ucp1* and *Cidea*, and promoted mitochondrial density in white glycolytic skeletal muscles and respiratory uncoupling (Chen et al. 2010). The regulatory roles of MED1 in major metabolic organs, such as liver, adipose tissues, and skeletal muscle, suggest that MED1 is an important regulator for metabolic gene expression. However, it is difficult to know whether the function(s) of MED1 are all mediator dependent or not.

### The tail module directly interacts with various transcription factors

The tail module includes subunits of MED14, MED15, MED16, MED23, MED24, and MED25. The metabolic roles of this module are well established, particularly for MED14, MED15, and MED23.

MED14 has been implicated in regulation of lipid homeostasis. It has been reported that MED14 binds to the N-terminal AF1 domain of GR (Hittelman et al. 1999) or PPARγ (Grontved et al. 2010) to activate target gene transcription. In vitro and in vivo assays show that MED14 directly interacts with GR, and this interaction increased GR-dependent transcription activation (Hittelman et al. 1999). In addition, MED14 directly interacts with PPARγ and promotes PPARγ-dependent transactivation as well as the recruitment of the Mediator complex (Grontved et al. 2010). Recently, it has been demonstrated that the Mediator complex is recruited to the PPARγ-target gene promoters without the LXXLL motifs of MED1, suggesting that MED14 may be more critically required for the transcription activation during adipogenesis (Grontved et al. 2010). Studies of MED14 knockdown revealed that the recruitment of PPARγ, MED6, MED8, and Pol II to the transcription start sites is dependent on MED14 in 3T3-L1 cells (Grontved et al. 2010). At present, it is unclear what the basis is for the apparent conflicting roles of MED1 and MED14 in the regulation of PPARγ-target genes. It remains to be established whether these differences reflect the different experimental interventions used, differences in cells examined, or differential interactions and physiologic outputs that occur during adipogenesis versus fully differentiated adipocytes. In addition, there may be undetermined cell-context effects resulting from the secondary transcription factor interactions. For example, MED14 was also reported to interact with the SREBP transcription factors (Toth et al. 2004).

The functions of MED15 and other mediator subunits in worms have been recently reviewed (Grants et al. 2015). In mammalian cells, MED15 acts as an important regulator of lipid biosynthesis through modulating the SREBPs (Yang et al. 2006). The interaction between MED15 and SREBPs is through the MED15-KIX domain, which is structurally similar to the KIX domains of CBP and p300 (Yang et al. 2006). Interestingly, the KIX domain of MED15 interacts only with SREBP-1a, but not with several other transcription factors examined, suggesting its binding specificity. The SREBP-target genes include the important lipogenic and cholesterogenic genes such as fatty acid synthase (*Fasn*) and HMG-CoA synthase (*Hmgcr*) (Amemiya-Kudo et al. 2002). By recruiting the Mediator complex upon binding to SREBPs, MED15 promotes SREBP-target gene expression (Naar et al. 1998, 1999). Interestingly, small-molecule inhibitors that block the interaction between the MED15-KIX domain and the transactivation domain of SREBP-1a protect mice from the metabolic dysregulation that occurs during diet-induced obesity (Zhao et al. 2014).

MED23 has been well studied in mammalian model systems. The importance of MED23 in viability has been shown with the embryonic lethality when MED23 was knocked out in mice (Balamotis et al. 2009). MED23 has been linked to insulin signaling in the adipogenesis transcription cascade (Wang et al. 2009). In 3T3-L1 cells, it has been shown that the interaction of MED23 with Elk1 is enhanced by the insulin-induced MAPK activation, resulting in further induction of Krox20, the initial transcription factor in the adipogenesis pathway (Wang et al. 2009). Interestingly, a recent study has shown that MED23 is also involved in regulating the differentiation of mesenchymal stem cells into smooth muscle cells or adipocytes (Yin et al. 2012). MED23 deficiency promoted mesenchymal stem cells into smooth muscle cells while preventing the differentiation into adipocytes (Yin et al. 2012). The mechanism by which MED23 controls the differentiation is via regulating the balance of serum response factor (SRF) downstream genes, *RhoA/MAL* or *Ras/ELK1*, by directly interacting with them in response to upstream signals (Stevens et al. 2002; Wang et al. 2009; Yin et al. 2012). MED23 favors the ELK1–SRF complex formation, which in turn facilitates the growth-related and adipogenic genes and suppresses the cytoskeleton and smooth muscle gene transcription (Yin et al. 2012). Furthermore, another recent report revealed that liver-specific knockdown or knockout of MED23 significantly improved glucose and lipid metabolism, especially for mice that were fed a high-fat diet (Chu et al. 2014). Reduced FoxO1-target gene expression was found in MED23-deficient primary hepatocytes, demonstrating that the regulation of MED23 on gluconeogenic gene expression is through FoxO1 (Chu et al. 2014). Regulation of the balance between adipocyte and smooth muscle development as well as hepatic lipid/glucose metabolism by MED23 suggests the importance of this subunit in metabolism.

It is known that HNF4α regulates a various set of genes that are not only involved in early development but also in liver and pancreatic cell differentiation, glucose metabolism, and lipid homeostasis (Odom et al. 2004). In yeast two-hybrid assays, MED25 was found to bind to HNF4α, inducing HNF4α-dependent gene transcription that maintains the normal function of insulin secretion in pancreatic β-cells (Odom et al. 2004). As a subunit in the tail module, MED25 interaction with HNF4α recruits the Mediator complex and Pol II for transcriptional activation (Rana et al. 2011). Although the exact mechanism is still unclear, these functional studies also suggest a role of MED25 in metabolism and lipid homeostasis.

### The kinase module functions in a context-dependent manner

Originally considered as a part of a transcriptional repressor in the large Mediator complex, the kinase module can repress or activate gene expression through kinase-dependent or kinase-independent mechanisms (Malik and Roeder 2010). Interestingly, the small mediator subunit MED26 is present in a mutually exclusive manner with CDK8 (Taatjes et al. 2002). Moreover, the kinase activity of CDK8 is not always required for its function in gene expression (Holstege et al. 1998). Besides the four conserved subunits in this module, paralogues have been identified for CDK8, MED12, and MED13, *i*.e., CDK19, MED12L, and MED13L (Daniels et al. 2013). Although their biological functions are less clear, these paralogues are subunits of the mammalian Mediator complex in a mutually exclusive manner with CDK8, MED12, and MED13 (Daniels et al. 2013). Among the four subunits, the evolutionarily conserved CDK8 and CycC have been studied for their functions in lipogenic gene expression (Zhao et al. 2012). The tissue-specific CDK19 is highly similar to CDK8 in amino acid sequence, but they may have both overlapping and distinct functions (Tsutsui et al. 2011). For instance, CDK8 but not CDK19 regulates HIF1-dependent gene expression (Galbraith et al. 2013). Recent studies also revealed the functional roles of MED12, MED13, and MED13L in cardiovascular and systemic metabolic regulation in both physiological and pathophysiological states.

The CDK8–CycC dimer negatively regulates de novo lipogenesis by reducing nuclear SREBP-1a or SREBP-1c protein stability (Zhao et al. 2012). SREBP-1a and SREBP-1c are key regulators of de novo lipogenesis, and posttranslational modifications represent critical mechanisms that regulate their activity and/or abundance (Brown and Goldstein 2008). In addition to acetylation/deacetylation and ubiquitination, the phosphorylation of nuclear forms of SREBP-1 is critical for the proteasome-mediated degradation (Sundqvist et al. 2005). Therefore, the direct phosphorylation of threonine 426 or 402 in SREBP-1a and SREBP-1c isoforms, respectively, by CDK8 is important to understand the mechanism of SREBP-1 regulation of lipid metabolism (Zhao et al. 2012). The functions of CDK8–CycC dimer may be independent of the Mediator complex, as up to 30% of CDK8 exists as a free form from the Mediator complex although the presence of MED12 is required for maximal kinase activity of CDK8 (Knuesel et al. 2009b). Interestingly, insulin stimulation in vitro and in vivo can reduce the protein levels of CDK8 and CycC (Zhao et al. 2012). In mouse livers, CDK8 knockdown increased the blood lipid levels and NAFLD-like phenotypes (Zhao et al. 2012). Recent data have further suggested that mTORC1 activation upon feeding or in states of insulin resistance or NAFLD is responsible for the down-regulation of CDK8 and CycC in the liver (Feng et al. 2015).

In addition to CDK8 function in the negative regulation of de novo lipogenesis through its kinase activity, it has been shown that CDK8 has a positive role in response to serum, likely at the elongation step instead of the initiation step where CDK8 is a negative regulator (Donner et al. 2010) (see (Nemet et al. 2014) for a recent review). The difficulty of identification of CDK8 substrates in vivo lies with embryonic lethality of CDK8-deficient mice, whereas there is no effect on cell viability in some cultured cells (Westerling et al. 2007). Nevertheless, using a selective inhibitor of CDK8 and CDK19 in tissue culture, a recent study has added more potential substrates of CDK8 and/or CDK19 to the increasing list (Poss et al. 2016), which includes Cyclin H (Akoulitchev et al. 2000), Notch (Fryer et al. 2004), histone H3 (Knuesel et al. 2009b), Smads (Alarcon et al. 2009), SREBP-1 (Zhao et al. 2012), E2F1 (Zhao et al. 2013), and STAT1 (Bancerek et al. 2013; Putz et al. 2013). However, further studies are necessary to understand the roles of CDK8 and CDK19 in metabolic regulation.

Through MED13, the MED12–MED13 dimer links the small mediator as well as the CDK8–CycC dimer (Tsai et al. 2013). Although the mechanism is unclear, MED12 can activate the Mediator complex-independent kinase activity of CDK8 in vitro (Knuesel et al. 2009b). Interestingly, MED13 in the heart regulates a subset of nuclear hormone receptor target genes that are major determinants of the metabolic rate and whole-body energy expenditure (Grueter et al. 2012). MED13 overexpression in the heart resulted in an increase in energy expenditure and resistance to diet-induced obesity (Grueter et al. 2012). MED13 is a target of miR-208a, one of the several microRNAs that are encoded by the cardiac-specific α-myosin heavy chain gene intron and are involved in heart disease and metabolic regulation (Montgomery et al. 2011; Grueter et al. 2012). Inhibition of miR-208a resulted in a similar metabolic phenotype that agrees with the finding that miR-208a negatively regulates MED13 (Grueter et al. 2012). Elevated protein levels of MED13 in the heart resulted in a significant increase in oxygen consumption, whereas the cardiac deletion of MED13 resulted in increased lipid accumulation without changes in food intake in mice (Grueter et al. 2012). MED13, but not MED13L, controls the whole-body metabolic homeostasis through altering metabolic profiles in white adipose tissue and liver when MED13 is altered in the heart (Baskin et al. 2014). Moreover, cardiac overexpression of MED13 in mice improves dysregulation of energy metabolism under high-fat diet likely through unknown circulating factors, as determined by heterotypic parabiosis experiments (Baskin et al. 2014). However, MED13 knockout in skeletal muscle resulted in resistance to hepatic steatosis in mice by activating a metabolic gene program that enhances muscle glucose uptake and storage as glycogen (Amoasii et al. 2016). Mechanistically, MED13 suppresses the expression of genes involved in glucose uptake and metabolism in skeletal muscle by inhibiting the nuclear receptor NURR1 and the MEF2 transcription factor (Amoasii et al. 2016). Although the roles of MED13 in other metabolic tissues have not been reported, the opposing metabolic regulation by MED13 in skeletal muscle and the heart further demonstrates the tissue-specific functions of the Mediator complex.

To date, not much information is available on the functions of MED12L and MED13L. Expressed in both heart and brain, MED13L is associated with early development of both heart and brain since its missense mutations and gene interruption are found in patients with congenital heart defect, learning disabilities, and facial anomalies (Muncke et al. 2003; Asadollahi et al. 2013; Davis et al. 2013). Both MED13 and MED13L are degraded by the SCF/Fbw7-dependent ubiquitination mechanism, and similar to MED13, MED13L may be also responsible for linking the kinase module to the small Mediator complex (Davis et al. 2013).

## Conclusion

Since the discovery of the Mediator complex as a transcription cofactor of eukaryotes, its subunits have been associated with various biological processes and several diseases ranging from developmental defects to cancer in animal models and humans. The metabolic functions of the Mediator complex have become increasingly significant. In most cases, the Mediator complex functions as a bridge to connect and integrate specific transcription factors to the basal transcription machinery, resulting in expression changes of a selective set of genes. In addition to the presence of many subunits, tissue-specific expression and possible diverse assembly states at different physiological conditions further complicate the biological functions of the Mediator complex. Future studies on the role of the Mediator complex in metabolism may include identification of how the metabolic or nutrient signals may regulate the assembly states and subunit compositions and investigation of tissue-specific functions of each subunit in regulating nutrient and energy metabolism in vivo.

## Abbreviations

Pol II: RNA polymerase II
T2D: Type 2 diabetes
NAFLD: Non-alcoholic fatty liver disease
CREB: cAMP-regulated enhancer-binding protein
FoxO: Forkhead O box protein
GR: Glucocorticoid receptor
HNF: Hepatic nuclear factor
SREBP: Sterol response element binding protein
LXR: Liver X receptor
PPAR: Peroxisome proliferator-activated receptor
CDK: Cyclin-dependent kinase
CycC: Cyclin C
ER: Estrogen receptor
CAR: Constitutive androstane receptor
UCP1: Uncoupling protein 1
SRC: Steroid receptor co-activator
BAT: Brown adipose tissue
SRF: Serum response factor
